# 4-Bromo­phenyl benzoate

**DOI:** 10.1107/S1600536808008167

**Published:** 2008-03-29

**Authors:** B. Thimme Gowda, Sabine Foro, K. S. Babitha, Hartmut Fuess

**Affiliations:** a Department of Chemistry, Mangalore University, Mangalagangotri 574 199, Mangalore, India; b Institute of Materials Science, Darmstadt University of Technology, Petersenstrasse 23, Darmstadt, D-64287, Germany

## Abstract

The structure of the title compound (4BPBA), C_13_H_9_BrO_2_, is similar to that of phenyl benzoate (PBA), 4-methyl­phenyl benzoate (4MePBA) and 4-methoxy­phenyl benzoate, with somewhat different bond parameters. The dihedral angle between the phenyl and benzoyl rings in 4BPBA is 58.43 (17)°, compared with values of 55.7° in PBA and 60.17 (7)° in 4MPBA. The mol­ecules in the title compound are packed into infinite chains in the *a*-axis direction.

## Related literature

For related literature, see: Adams & Morsi (1976 ▶); Gowda, Foro, Babitha & Fuess (2007 ▶); Gowda, Foro, Nayak & Fuess (2007 ▶); Nayak & Gowda (2008 ▶).
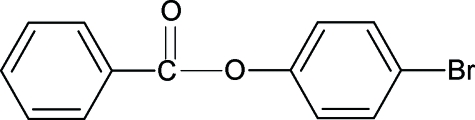


## Experimental

### 

#### Crystal data

C_13_H_9_BrO_2_
*M* *_r_* = 277.11
Orthorhombic, 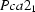

*a* = 7.748 (1) Å
*b* = 5.5946 (7) Å
*c* = 26.814 (5) Å
*V* = 1162.3 (3) Å^3^
*Z* = 4
Cu *K*α radiation
μ = 4.67 mm^−1^
*T* = 299 (2) K
0.38 × 0.30 × 0.08 mm

#### Data collection

Enraf–Nonius CAD-4 diffractometer
Absorption correction: ψ scan (North *et al.*, 1968 ▶) *T*_min_ = 0.241, *T*_max_ = 0.685
1986 measured reflections
1442 independent reflections
1252 reflections with *I* > 2σ(*I*)
*R*_int_ = 0.039
3 standard reflections frequency: 120 min intensity decay: 2.0%

#### Refinement

*R*[*F* ^2^ > 2σ(*F* ^2^)] = 0.059
*wR*(*F* ^2^) = 0.189
*S* = 1.15
1442 reflections
145 parameters
1 restraint
H-atom parameters constrained
Δρ_max_ = 1.04 e Å^−3^
Δρ_min_ = −1.32 e Å^−3^
Absolute structure: Flack (1983 ▶), with 375 Friedel pairs
Flack parameter: −0.04 (6)

### 

Data collection: *CAD-4-PC Software* (Enraf–Nonius, 1996 ▶); cell refinement: *CAD-4-PC Software*; data reduction: *REDU4* (Stoe & Cie, 1987 ▶); program(s) used to solve structure: *SHELXS97* (Sheldrick, 2008 ▶); program(s) used to refine structure: *SHELXL97* (Sheldrick, 2008 ▶); molecular graphics: *PLATON* (Spek, 2003 ▶); software used to prepare material for publication: *SHELXL97*.

## Supplementary Material

Crystal structure: contains datablocks I, global. DOI: 10.1107/S1600536808008167/om2221sup1.cif

Structure factors: contains datablocks I. DOI: 10.1107/S1600536808008167/om2221Isup2.hkl

Additional supplementary materials:  crystallographic information; 3D view; checkCIF report

## Figures and Tables

**Fig. 1.**
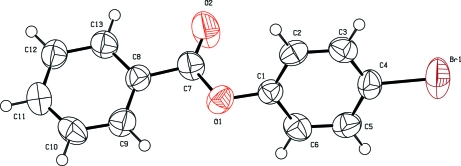
Molecular structure of the title compound, showing the atom labeling. Displacement ellipsoids are drawn at the 50% probability level.

**Fig. 2.**
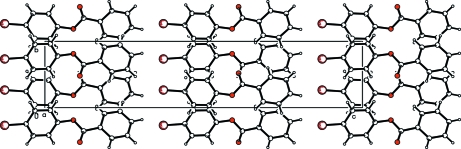
Molecular packing of the title compound as viewed down the bc plane.

## supplementary crystallographic information

### Comment

In the present work, the structure of 4-bromophenyl benzoate (4BPBA) has been determined, as part of a study of substituent effects on the structures of industrially significant compounds (Gowda, Foro, Babitha & Fuess, 2007; Gowda, Foro, Nayak & Fuess, 2007). The structure of 4BPBA (Fig. 1) resembles those of phenyl benzoate (PBA) (Adams & Morsi, 1976), 4-methylphenyl benzoate (4MePBA), 4-methoxyphenyl benzoate (4MeOPBA), 3-methylphenyl benzoate (3MePBA), 2,3-dichlorophenyl benzoate (23DCPBA) and other aryl benzoates (Gowda, Foro, Babitha & Fuess, 2007; Gowda, Foro, Nayak & Fuess, 2007). The bond parameters in 4BPBA are similar to those in PBA, 4MePBA, 4MeOPBA, 3MePBA, 23DCPBA and other benzoates (Adams & Morsi, 1976; Gowda, Foro, Babitha & Fuess, 2007; Gowda, Foro, Nayak & Fuess, 2007). The molecules in the title compound are packed into chains in the *bc* plane (Fig. 2).

### Experimental

The title compound was prepared according to a literature method (Nayak & Gowda, 2008). The purity of the compound was checked by determining its melting point. It was characterized by recording its infrared and NMR spectra (Nayak & Gowda, 2008). Single crystals of the title compound were obtained by slow evaporation of an ethanolic solution and used for X-ray diffraction studies at room temperature.

### Refinement

The H atoms were positioned with idealized geometry using a riding model (C—H = 0.93 Å) with *U*_iso_ = 1.2 *U*_eq_ of the parent atom.

The residual electron-density features are located in the region of Br1. The highest peak is 0.91 Å from C4 and deepest hole is 0.78 Å from Br1.

### Crystal data

**Table:**

C_13_H_9_BrO_2_	*F*_000_ = 552
*M**_r_* = 277.11	*D*_x_ = 1.584 Mg m^−^^3^
Orthorhombic, *P**c**a*2_1_	Cu *K*α radiation λ = 1.54180 Å
Hall symbol: P 2c -2ac	Cell parameters from 25 reflections
*a* = 7.748 (1) Å	θ = 9.9–23.4º
*b* = 5.5946 (7) Å	µ = 4.67 mm^−^^1^
*c* = 26.814 (5) Å	*T* = 299 (2) K
*V* = 1162.3 (3) Å^3^	Plate, colourless
*Z* = 4	0.38 × 0.30 × 0.08 mm

### Data collection

**Table:**

Enraf–Nonius CAD-4 diffractometer	*R*_int_ = 0.039
Radiation source: fine-focus sealed tube	θ_max_ = 66.9º
Monochromator: graphite	θ_min_ = 3.3º
*T* = 299(2) K	*h* = −1→9
ω/2θ scans	*k* = −1→6
Absorption correction: ψ scan(North *et al.*, 1968)	*l* = −8→32
*T*_min_ = 0.242, *T*_max_ = 0.685	3 standard reflections
1986 measured reflections	every 120 min
1442 independent reflections	intensity decay: 2.0%
1252 reflections with *I* > 2σ(*I*)	

### Refinement

**Table:**

Refinement on *F*^2^	Hydrogen site location: inferred from neighbouring sites
Least-squares matrix: full	H-atom parameters constrained
*R*[*F*^2^ > 2σ(*F*^2^)] = 0.059	*w* = 1/[σ^2^(*F*_o_^2^) + (0.1268*P*)^2^ + 0.565*P*] where *P* = (*F*_o_^2^ + 2*F*_c_^2^)/3
*wR*(*F*^2^) = 0.189	(Δ/σ)_max_ = 0.001
*S* = 1.15	Δρ_max_ = 1.04 e Å^−^^3^
1442 reflections	Δρ_min_ = −1.32 e Å^−^^3^
145 parameters	Extinction correction: none
1 restraint	Absolute structure: Flack (1983), with 375 Friedel pairs
Primary atom site location: structure-invariant direct methods	Flack parameter: −0.04 (6)
Secondary atom site location: difference Fourier map	

### Special details

**Table:**

Geometry. All e.s.d.'s (except the e.s.d. in the dihedral angle between two l.s. planes) are estimated using the full covariance matrix. The cell e.s.d.'s are taken into account individually in the estimation of e.s.d.'s in distances, angles and torsion angles; correlations between e.s.d.'s in cell parameters are only used when they are defined by crystal symmetry. An approximate (isotropic) treatment of cell e.s.d.'s is used for estimating e.s.d.'s involving l.s. planes.
Refinement. Refinement of *F*^2^ against ALL reflections. The weighted *R*-factor *wR* and goodness of fit *S* are based on *F*^2^, conventional *R*-factors *R* are based on *F*, with *F* set to zero for negative *F*^2^. The threshold expression of *F*^2^ > σ(*F*^2^) is used only for calculating *R*-factors(gt) *etc*. and is not relevant to the choice of reflections for refinement. *R*-factors based on *F*^2^ are statistically about twice as large as those based on *F*, and *R*- factors based on ALL data will be even larger.

### Fractional atomic coordinates and isotropic or equivalent isotropic displacement parameters (Å^2^)

**Table:**

	*x*	*y*	*z*	*U*_iso_*/*U*_eq_	
Br1	0.29159 (16)	0.2659 (2)	−0.12742 (8)	0.0950 (5)	
O1	0.4692 (6)	0.1882 (9)	0.0901 (2)	0.0581 (13)	
O2	0.3231 (11)	0.5195 (14)	0.1104 (3)	0.107 (3)	
C1	0.4260 (8)	0.2190 (10)	0.0403 (3)	0.0463 (14)	
C2	0.4854 (8)	0.4121 (12)	0.0127 (3)	0.0544 (16)	
H2	0.5500	0.5321	0.0278	0.065*	
C3	0.4478 (8)	0.4229 (12)	−0.0367 (3)	0.0560 (17)	
H3	0.4888	0.5492	−0.0559	0.067*	
C4	0.3474 (11)	0.2440 (12)	−0.0586 (3)	0.0559 (17)	
C5	0.2920 (9)	0.0437 (12)	−0.0313 (3)	0.0589 (18)	
H5	0.2301	−0.0794	−0.0462	0.071*	
C6	0.3332 (9)	0.0389 (13)	0.0176 (3)	0.0583 (17)	
H6	0.2977	−0.0908	0.0368	0.070*	
C7	0.4106 (9)	0.3510 (14)	0.1226 (3)	0.0577 (17)	
C8	0.4601 (9)	0.2976 (12)	0.1748 (3)	0.0521 (15)	
C9	0.5527 (8)	0.0900 (13)	0.1879 (3)	0.0585 (17)	
H9	0.5846	−0.0209	0.1638	0.070*	
C10	0.5943 (10)	0.0558 (13)	0.2368 (4)	0.0644 (19)	
H10	0.6544	−0.0820	0.2454	0.077*	
C11	0.5530 (10)	0.2107 (14)	0.2731 (4)	0.0627 (19)	
H11	0.5834	0.1803	0.3060	0.075*	
C12	0.4636 (9)	0.4180 (14)	0.2604 (3)	0.0602 (17)	
H12	0.4353	0.5284	0.2851	0.072*	
C13	0.4164 (9)	0.4607 (13)	0.2112 (3)	0.0585 (16)	
H13	0.3558	0.5985	0.2029	0.070*	

### Atomic displacement parameters (Å^2^)

**Table:**

	*U*^11^	*U*^22^	*U*^33^	*U*^12^	*U*^13^	*U*^23^
Br1	0.1056 (8)	0.1322 (9)	0.0472 (6)	0.0167 (6)	−0.0058 (7)	−0.0056 (7)
O1	0.053 (2)	0.064 (3)	0.058 (3)	0.008 (2)	−0.003 (2)	−0.011 (3)
O2	0.145 (6)	0.117 (5)	0.059 (4)	0.089 (5)	0.005 (4)	0.001 (4)
C1	0.044 (3)	0.047 (3)	0.049 (4)	0.003 (2)	−0.001 (3)	−0.003 (3)
C2	0.046 (3)	0.052 (3)	0.066 (5)	−0.007 (3)	0.004 (3)	−0.009 (3)
C3	0.055 (3)	0.052 (3)	0.061 (5)	0.007 (3)	0.011 (3)	0.005 (3)
C4	0.058 (4)	0.067 (4)	0.042 (4)	0.006 (3)	0.005 (4)	−0.006 (3)
C5	0.062 (4)	0.049 (3)	0.066 (5)	−0.004 (3)	−0.005 (4)	−0.006 (3)
C6	0.054 (3)	0.058 (4)	0.063 (5)	−0.012 (3)	0.002 (4)	0.010 (4)
C7	0.050 (3)	0.068 (4)	0.055 (4)	0.019 (3)	0.004 (3)	0.002 (4)
C8	0.051 (3)	0.055 (3)	0.050 (4)	−0.005 (3)	0.006 (3)	−0.001 (3)
C9	0.053 (3)	0.062 (3)	0.061 (4)	0.017 (3)	−0.004 (3)	−0.010 (4)
C10	0.057 (3)	0.062 (4)	0.074 (5)	0.007 (3)	−0.008 (4)	0.015 (4)
C11	0.056 (3)	0.082 (5)	0.050 (5)	−0.001 (3)	−0.002 (3)	0.003 (4)
C12	0.064 (4)	0.065 (4)	0.052 (4)	−0.007 (3)	0.005 (4)	−0.002 (4)
C13	0.056 (3)	0.059 (4)	0.060 (4)	0.010 (3)	−0.003 (3)	0.001 (3)

### Geometric parameters (Å, °)

**Table:**

Br1—C4	1.899 (9)	C6—H6	0.9300
O1—C7	1.340 (10)	C7—C8	1.481 (12)
O1—C1	1.388 (10)	C8—C13	1.379 (11)
O2—C7	1.206 (9)	C8—C9	1.410 (10)
C1—C6	1.379 (10)	C9—C10	1.364 (12)
C1—C2	1.388 (10)	C9—H9	0.9300
C2—C3	1.356 (12)	C10—C11	1.342 (12)
C2—H2	0.9300	C10—H10	0.9300
C3—C4	1.398 (11)	C11—C12	1.392 (11)
C3—H3	0.9300	C11—H11	0.9300
C4—C5	1.406 (11)	C12—C13	1.390 (12)
C5—C6	1.351 (12)	C12—H12	0.9300
C5—H5	0.9300	C13—H13	0.9300
			
C7—O1—C1	117.4 (5)	O2—C7—C8	124.1 (7)
C6—C1—C2	120.4 (8)	O1—C7—C8	112.9 (6)
C6—C1—O1	117.3 (6)	C13—C8—C9	119.5 (8)
C2—C1—O1	122.0 (6)	C13—C8—C7	118.2 (6)
C3—C2—C1	118.9 (6)	C9—C8—C7	122.2 (7)
C3—C2—H2	120.5	C10—C9—C8	118.4 (7)
C1—C2—H2	120.5	C10—C9—H9	120.8
C2—C3—C4	119.9 (7)	C8—C9—H9	120.8
C2—C3—H3	120.1	C11—C10—C9	123.4 (7)
C4—C3—H3	120.1	C11—C10—H10	118.3
C3—C4—C5	121.4 (8)	C9—C10—H10	118.3
C3—C4—Br1	119.3 (6)	C10—C11—C12	118.7 (8)
C5—C4—Br1	119.2 (6)	C10—C11—H11	120.7
C6—C5—C4	116.7 (7)	C12—C11—H11	120.7
C6—C5—H5	121.6	C13—C12—C11	120.4 (8)
C4—C5—H5	121.6	C13—C12—H12	119.8
C5—C6—C1	122.4 (7)	C11—C12—H12	119.8
C5—C6—H6	118.8	C8—C13—C12	119.6 (7)
C1—C6—H6	118.8	C8—C13—H13	120.2
O2—C7—O1	123.0 (8)	C12—C13—H13	120.2
			
C7—O1—C1—C6	−120.1 (7)	C1—O1—C7—C8	178.5 (6)
C7—O1—C1—C2	65.4 (9)	O2—C7—C8—C13	−5.8 (12)
C6—C1—C2—C3	1.3 (10)	O1—C7—C8—C13	175.9 (6)
O1—C1—C2—C3	175.6 (6)	O2—C7—C8—C9	175.6 (9)
C1—C2—C3—C4	1.4 (10)	O1—C7—C8—C9	−2.7 (10)
C2—C3—C4—C5	−3.8 (10)	C13—C8—C9—C10	0.9 (10)
C2—C3—C4—Br1	178.5 (5)	C7—C8—C9—C10	179.5 (7)
C3—C4—C5—C6	3.2 (10)	C8—C9—C10—C11	−0.6 (12)
Br1—C4—C5—C6	−179.1 (6)	C9—C10—C11—C12	−0.3 (12)
C4—C5—C6—C1	−0.5 (11)	C10—C11—C12—C13	0.9 (11)
C2—C1—C6—C5	−1.8 (11)	C9—C8—C13—C12	−0.3 (11)
O1—C1—C6—C5	−176.4 (7)	C7—C8—C13—C12	−178.9 (7)
C1—O1—C7—O2	0.2 (12)	C11—C12—C13—C8	−0.6 (11)
